# Neuroethology of reward and decision making

**DOI:** 10.1098/rstb.2008.0159

**Published:** 2008-10-01

**Authors:** Karli K Watson, Michael L Platt

**Affiliations:** 1 Department of Neurobiology, Duke UniversityDurham, NC 27708, USA; 2 Center for Neuroeconomic StudiesDuke University, Durham, NC 27708, USA; 3 Center for Cognitive Neuroscience, Duke UniversityDurham, NC 27708, USA; 4 Department of Biological Anthropology and Anatomy, Duke UniversityDurham, NC 27708, USA

**Keywords:** foraging, game theory, neuroeconomics, neuroethology, risk, social reward

## Abstract

Ethology, the evolutionary science of behaviour, assumes that natural selection shapes behaviour and its neural substrates in humans and other animals. In this view, the nervous system of any animal comprises a suite of morphological and behavioural adaptations for solving specific information processing problems posed by the physical or social environment. Since the allocation of behaviour often reflects economic optimization of evolutionary fitness subject to physical and cognitive constraints, neurobiological studies of reward, punishment, motivation and decision making will profit from an appreciation of the information processing problems confronted by animals in their natural physical and social environments.

## 1. Introduction

The unifying goal of ethology, as well as the newer fields of behavioural ecology and sociobiology, is to provide evolutionary explanations for behaviour (Hinde 1982; Krebs & Davies 1993; Trivers 2002). This approach proposes that the forces of natural and sexual selection favour behaviours that maximize the reproductive success of individuals within the context of their native physical and social environments. Ethologically, rewards can be considered proximate goals that, when acquired, tend to enhance survival and mating success (Hinde 1982). Similarly, avoiding punishment is a proximate goal that ultimately serves to augment the long-term likelihood of survival and reproduction. These assumptions thus extend the traditional psychological and neurobiological notions of reward and punishment, which are typically defined by the quality of eliciting approach and avoidance, respectively (Skinner 1938; Robbins & Everitt 1996). As detailed below, the assumption of evolutionary adaptation within ethology, behavioural ecology and sociobiology has promoted the development of mathematical models that formally define rewards and punishments within specific behavioural contexts. Such models imply that full understanding of the neurobiology of reward and decision making will require consideration of naturally occurring behaviours in the specific ecological and social contexts in which they are normally expressed.

## 2. The economics of foraging behaviour

One of the most fundamental choices an animal must confront while foraging is to decide between exploitation and exploration, i.e. whether to consume what is at hand or to search for better alternatives. Optimal foraging theory represented an early application of economic modelling to animal foraging behaviour to derive the theoretical ‘optimal’ solution to such dilemmas (see Stephens & Krebs (1986) for a review). One of the first models was developed by MacArthur & Pianka (1966) who defined the criteria for the consumption or rejection of prey items associated with different levels of energetic investment (to hunt or otherwise procure) and different rates of energetic return. This ‘prey model’ begins with the premise that the average rate of energy intake R may be modelled as the ratio of the energetic benefit afforded to the animal relative to the time costs of foraging

$$R=\frac{E}{T_{\text{h}}+T_{\text{s}}},$$

where *R* is the net benefit gained by the predator for consuming a particular prey type; *E* is the amount of energy gained; *T*_h_ is the handling time; and *T*_s_ is the search time. The model is solved to maximize *R*, which determines the diet offering the greatest net energetic return and thus maximizing evolutionary success.

One prediction of this model is that the greater the abundance of higher quality foods, the less an animal's diet will consist of lower quality foods (the so-called ‘independence of inclusion from encounter’ rule). Goss-Custard (1977) found evidence in support of this prediction in his studies of redshanks, small wading birds, found in estuarine habitats in Great Britain. Redshanks feed on crustaceans, which offer higher energetic returns, and worms, which offer lower energetic returns. As predicted, the birds did not indiscriminately consume every worm or crustacean encountered; instead, they exclusively ate crustaceans when their density was high, but included worms in the diet when crustacean density declined. This result makes intuitive sense because the cost of eating worms includes missed opportunities to search for and eat more nutritionally profitable crustaceans. When crustaceans are rare, however, it is more profitable to focus on small but abundant worms rather than wasting time searching for higher value foods.

The behaviour of a wide variety of species, including birds (Davies 1977; Goss-Custard 1977), spiders (Diaz-Fleischer 2005), fishes (Anderson 1984) and even humans (Milton 1979; Waddington & Holden 1979; Hawkes *et al*. 1982), has been found to fit the general predictions of the prey model. However, as is often the case in economics, MacArthur and Pianka's simple model does not perfectly describe behaviour in the real world. While the formal mathematical derivation of the prey model predicts a step-like change in preference, in which one type of prey is always preferred to the exclusion of the other (the ‘zero–one rule’), the birds in Goss-Custard's study showed ‘partial preferences’ for different types of prey (e.g. preferring one type 75% of the time) when their density changed. Such partial preferences might reflect sampling behaviour, which allows the animal to acquire improved information about the statistics of the local environment (Krebs *et al*. 1977). Alternatively, partial preferences could reflect sensory- or memory-related cognitive limitations that interfere with the expression of optimal behaviour (Stephens & Krebs 1986).

In addition to selecting between prey items, animals that forage for foods that are clumped in space and time must decide how long to spend foraging within a particular patch before abandoning it and moving onto another. Charnov's (1976) marginal value theorem models a forager's behaviour given a patchy distribution of resources. Here the fundamental decision is whether to spend more time searching for prey in a given patch, or whether to switch to a new patch, which requires both time and energy. As in the prey model, the patch model assumes that the forager's goal is to maximize *R*, the average rate of energy intake. By definition, individual patches in the environment have finite resources, which the forager will eventually deplete. Charnov deduced that an animal foraging in a patchy environment should leave the patch when the rate of gain from that patch is equal to the overall rate of gain from the environment as a whole. This prediction is easily tested by measuring the time elapsed before the animal leaves its current patch in search of a new one, and has been upheld in several different experimental paradigms with several species (Cowie 1977; Lima 1983).

**Figure 1:**
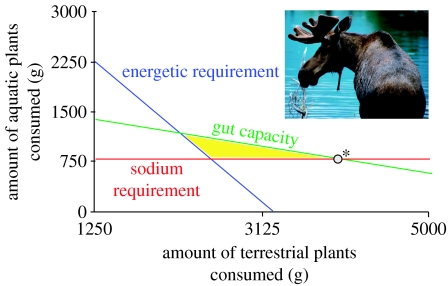
Optimal diet choice in elk (*Alces alces*). The daily ratio of aquatic to terrestrial plants consumed by elk must satisfy three constraints: the diet must meet energetic (blue line) and sodium requirements (red line), subject to digestive limitations (green line). Those ratios that meet these constraints are contained in the yellow-shaded area. The vertex marked with an asterisk indicates the aquatic-to-terrestrial plant ratio that maximizes energetic intake while also satisfying all constraints. Adapted from Stephens & Krebs (1986). Photo courtesy of the National Park Service.

Early models, including the patch and prey models, described behavioural optimization in a generic sense, without regard to the specific physiological or cognitive constraints on a particular animal. Making precise predictions for individuals of a given species, on the other hand, requires consideration of the specific physiological and environmental constraints on that animal. For example, elk foraging for terrestrial and aquatic plants must satisfy both energetic needs and sodium requirements within the limitations imposed by gut capacity. Terrestrial plants are richer in energy than aquatic plants, and take up less room in the gut. However, aquatic plants contain more sodium than terrestrial plants, and, because aquatic plants are buried under ice during the winter, elk must consume enough of them during the summer to satisfy their sodium requirements for the rest of the year (Belovsky 1978). According to the model, elk can maximize energetic returns while simultaneously satisfying sodium needs and rumen constraints by selecting a diet comprising 18 per cent aquatic plants and 82 per cent terrestrial plants—in precise agreement with the observations of foraging elk in the wild (figure 1).

Early field studies testing optimal foraging models, such as Goss-Custard's redshank study and Belovsky's elk study, demonstrated the strengths of the economic approach: formulation of models allows for clear and precise predictions that can be tested empirically, and provides a quantitative tool around which to organize explanations of behaviour. Importantly for this review, such models make clear that defining rewards and punishments requires careful consideration of the behavioural and physiological capacities of a given species and the specific physical and social environments in which they normally act. The same resource may be pursued as a ‘reward’ in some contexts and avoided in others.

## 3. Neurobiology of reward and decision making

Ultimately, the nervous systems of humans and other animals have evolved to promote behaviours that enhance fitness, such as acquiring food and shelter, attracting mates, avoiding predators and prevailing over competitors. To achieve these goals, animal brains have become exquisitely specialized to attend to important features of the environment, extract their predictive value for success or failure and then use this information to compute the evolutionarily optimal course of action. Traditionally, these brain mechanisms have been studied with regard to their roles in acquiring rewards and avoiding punishments.

As noted above, rewards are traditionally defined as stimuli that elicit approach behaviour, while punishments can be defined as stimuli that elicit avoidance. Recent studies have revealed elementary properties of the neural systems that process rewards and punishments as traditionally defined. Specifically, the circuit connecting midbrain dopamine neurons to the ventral striatum and prefrontal cortex appears to be crucial for processing information about rewards (Schultz 2000; Schultz & Dickinson 2000). For example, animals will work to receive stimulation delivered via electrodes implanted in the dopaminergic ventral tegmental area (VTA), lateral hypothalamus or medial forebrain bundle, which connects the VTA to the ventral striatum (Olds & Milner 1954; Carlezon & Chartoff 2007). In fact, animals will preferentially work for such intracranial self-stimulation, to the exclusion of acquiring natural reinforcers such as food or water (Routtenberg & Lindy 1965; Frank & Stutz 1984).

**Figure 2:**
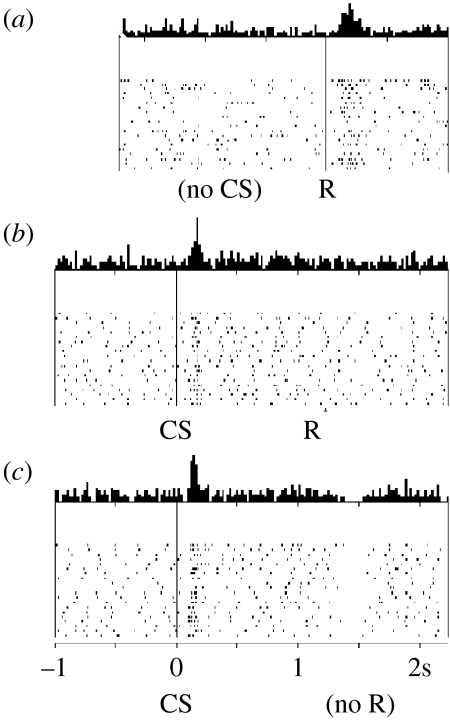
Reward-related responses by a single dopamine neuron recorded in a macaque monkey. (*a*) When the animal is still learning the task, the fruit juice reward is unexpected, and the neuron responds at the time of reward delivery (R) (no prediction, reward occurs). (*b*) After the monkey learns the relationship between a conditioned stimulus (such as a light or tone) and a reward, the neuron responds to the conditioned stimulus that predicts reward delivery (CS), but not to the reward itself (reward predicted, reward occurs). (*c*) If the reward is omitted after the predictive stimulus, dopamine neuron activity is suppressed during the time of expected reward delivery (reward predicted, no reward occurs). Each raster indicates the time of neuron spiking, and each row corresponds to a single trial for that neuron. The histograms summate the spikes over all the trials. Adapted from Schultz *et al*. (1997).

Electrophysiological recordings from dopaminergic neurons show that these cells respond to unpredicted primary rewards, such as food and water, as well as to conditioned stimuli that predict such rewards (Schultz 2000; Schultz & Dickinson 2000; figure 2). Moreover, dopamine neuron responses scale with both reward magnitude and reward probability (Fiorillo *et al*. 2003; Tobler *et al*. 2005). Dopamine neurons do not, however, merely signal rewards and the stimuli that predict them. Current evidence suggests that phasic bursts by dopamine neurons may correspond to the reward prediction error term initially proposed in purely behavioural models of learning (Schultz *et al*. 1997). According to this view, such phasic dopamine responses provide a mechanism for updating predicted valuation functions, which can be used both to learn about stimuli in the environment and to select profitable courses of action (Montague & Berns 2002). These valuation functions can be thought of as the neural implementation of the optimization functions assumed to guide behaviour in economic models developed in behavioural ecology.

**Figure 3:**
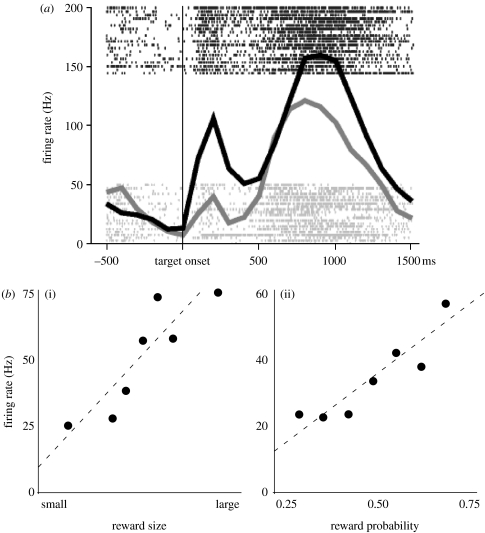
LIP neurons encode visual target value. (*a*) Neuronal firing is greater during trials when the expected reward is large (black line) than when it is small (grey line). Black and grey rasters indicate the time of individual spikes for large and small reward trials, respectively; each line of rasters corresponds to a single trial. Curves represent the summation of activity over all the trials. (*b*) Firing rate of a single LIP neuron increases linearly with (i) reward size and (ii) reward probability. Adapted from Platt & Glimcher (1999).

Signals from the dopaminergic midbrain neurons influence processing within decision-making areas, primarily orbital and medial prefrontal cortices, that assign value to sensory stimuli (Schultz *et al*. 2000). Value signals in these areas may inform processing in areas such as dorsolateral prefrontal and parietal cortices, which eventually transform that information into motor output (Gold & Shadlen 2001; Sugrue *et al*. 2004). For example, Platt & Glimcher (1999) probed the impact of expected value on sensory–motor processing in the lateral intraparietal (LIP) area, a region of the brain previously linked to visual attention and motor preparation. In that study, monkeys were cued to shift gaze from a central light to one of two peripheral lights to receive a fruit juice reward. In separate blocks of trials, the authors varied the expected value of orienting to each light by varying either the size of reward or the probability the monkey would be cued to shift gaze to each of the lights. Platt and Glimcher found that LIP neurons signalled target value, the product of reward size and saccade likelihood, prior to cue onset (figure 3). In a second experiment, monkeys were permitted to choose freely between the two targets, and both neuronal activity in the LIP area and the probability of target choice were correlated with target value.

Sugrue, Corrado and Newsome extended these observations by probing the dynamics of decision-related activity in the LIP area using a virtual foraging task (Sugrue *et al*. 2004). In that experiment, the rewards associated with each of two targets fluctuated over time. Under these conditions, monkeys tended to match the rate of choosing each target to its relative rate of reinforcement. Moreover, the responses of individual LIP neurons to a particular target corresponded to the relative rate of reward gained from choosing it on recent trials, with the greatest weight placed on the most recent trials. Together, these and other studies suggest that simple behavioural decisions may be computed by scaling neuronal responses associated with a particular stimulus or movement by its value, thus modifying the likelihood of reaching the threshold for eliciting a specific motor action (Gold & Shadlen 2001).

## 4. Uncertainty and decision making

Early ethological models of behaviour assumed that animals have complete knowledge of the environment and that reward contingencies are deterministic (Stephens & Krebs 1986). In practice, however, uncertainty about environmental contingencies places strong constraints on behaviour. The impact of uncertainty on choice has long been acknowledged in economics, which defines the spread of an outcome's known probability as risk. In the eighteenth century, Bernoulli (1954 (1738)) proposed that the expected values of monetary transactions, particularly risky financial ventures, differ from their corresponding subjective utilities (as determined by the economic agent). This idea, which would eventually revolutionize the field of economics, challenged the traditional notion that people value outcomes strictly according to their financial returns.

Initial economic models applied to animal behaviour explicitly ignored variance in reward outcomes. For example, Charnov's (1976) marginal value theorem, devised to predict when a foraging animal should leave a particular food patch, is based purely on the average distribution of resources among locations. Although this model predicts behaviour in simple contexts fairly well, it fails to account for behavioural sensitivity to variability within patches. Yet risk strongly determines how animals choose among available options (reviewed in Bateson & Kacelnik 1998), and the impact of risk on decision making itself can be influenced by behavioural context or internal state. For example, Caraco observed the behaviour of yellow-eyed juncos, a species of small songbirds native to Mexico and the southwestern United States (Caraco *et al*. 1980; Caraco 1981). The birds were given the option of choosing a tray with a fixed number of millet seeds or a tray with a probabilistically varying number of seeds with the same mean as the fixed option. Surprisingly, preferences depended on the ambient temperature. At 19°C juncos preferred the fixed option, but at 1°C they preferred the variable option. The proposed explanation for this switch from risk aversion to risk seeking is that, at the higher temperature, the rate of gain from the fixed option was sufficient to maintain the bird on a positive energy budget. At the lower temperature, however, energy expenditures were elevated, so the fixed option was no longer adequate to meet the animal's energy needs. When cold, the bird's best chance for survival was to gamble on the risky option since it might yield a higher rate of return than the fixed option. Energy budget has been reported to impact risk taking in a variety of animal species, including fishes (Young *et al*. 1990), insects (Cartar & Dill 1990) and mammals (Barnard *et al*. 1983; Ito *et al*. 2000), although the ubiquity of this relationship has been questioned (Kacelnik & Bateson 1996). This principle also appears to describe human choices in experiments using either money (Pietras *et al*. 2003) or opiates (Bickel *et al*. 2004) as a reward. These observations strongly suggest that sensitivity to risk is a widespread neural adaptation that evolved to support decision making.

Recent neurobiological studies have explored these neural mechanisms in both human and non-human primates (Glimcher 2003; Sanfey *et al*. 2006). In humans, preference for a risky option is associated with increases in neuronal activity in the ventral striatum and posterior parietal cortex (Huettel *et al*. 2006). Moreover, choosing a risky option activates the dorsal striatum, precuneus and premotor cortex (Hsu *et al*. 2005). A recent electrophysiological study in monkeys probed how such risk-related activity might inform action (McCoy & Platt 2005). Monkeys were given a choice between juice rewards of fixed or variable sizes with the same mean reward rate. Under these conditions, monkeys showed a strong preference for the risky option. Simultaneous recordings from single neurons in the posterior cingulate cortex, a region of the brain associated with spatial attention, visual orienting and reward processing, revealed that firing rates were correlated with subjective preferences for the risky option. Furthermore, spatial sensitivity of neurons in posterior cingulate was enhanced in riskier contexts. Such risk-induced changes in response gain may enhance the expression of strong behavioural preferences. The foregoing discussion makes plain that the discrepancy between observable outcomes and subjective preferences in decision making under risk offers a powerful paradigm for investigating the neural mechanisms underlying adaptive decision making.

## 5. Neural systems mediating exploration and exploitation

**Figure 4:**
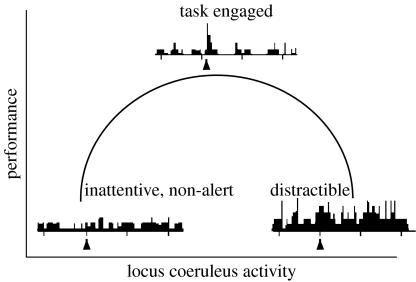
Neurons in the LC reflect the level of task engagement. A sleepy, uninterested monkey has a low level of baseline firing and no task-related phasic response in the LC (left). A monkey that is engaged in the task has low baseline firing paired with a phasic response that is linked to the motor actions performed in compliance with task demands (centre). An unfocused, distractible monkey has an attenuated phasic response coupled with high baseline firing (right). This mode of LC responsiveness may signal to the monkey that it is time to switch tasks. Arrowheads signify the onset of target stimuli. Adapted from Aston-Jones *et al*. (1999).

As formalized in Charnov's marginal value theorem, described above, an animal foraging in an environment with a heterogeneous distribution of resources must, at some point, choose to leave the current food patch to search for an alternative, potentially more rewarding patch. The locus coeruleus (LC), a collection of noradrenergic cells located in the pons, may mediate the shift from resource exploitation to exploration (Aston-Jones *et al*. 1999; Aston-Jones & Cohen 2005). These cells receive strong projections from the anterior cingulate (ACC) and orbitofrontal cortices (OFC), which may carry information about the current behavioural context and recent reward history (Aston-Jones & Cohen 2005). LC noradrenergic neurons, in turn, project diffusely throughout the brain. These projections appear to adjust the responsiveness of target structures to synaptic inputs (Foote *et al*. 1983; Aston-Jones *et al*. 1999). Recordings from monkeys indicate that LC neurons have moderate baseline activity punctuated by marked phasic responses linked to task-related cues and motor outputs. This pattern of activity is evident only when the animal is well engaged in the task at hand. When the animal is unfocused and distractible, however, as indicated by an increase in errors and failed trials, LC neurons switch from firing in the phasic mode to a tonic high level of firing. At the other extreme, when the monkey is sleepy and inattentive, LC neurons fire at low tonic rates with an absence of phasic bursts (Aston-Jones & Cohen 2005; figure 4).

Together, these qualities suggest that LC neurons might generate signals that trigger shifts in behavioural strategy. Decreasing marginal utility may be communicated to the LC by the ACC and the OFC, which may shift the LC between the phasic and tonic modes of activity (Aston-Jones *et al*. 1999; Aston-Jones & Cohen 2005). In one mode, phasic LC neuron firing is task related, and the animal persists its behaviour, presumably because the rewarding aspects of the task are greater than the associated cognitive and physiological demands. In the alternative mode, however, an increase in baseline firing reflects diminished utility derived from performing the task. This, in turn, frees up cognitive resources to switch to other, potentially more rewarding, behaviours. This interpretation implies that the depletion of resources in a particular resource patch may be encoded by the firing rates of neurons in the ACC and the OFC. Consistent with this idea, the ACC and the OFC are active during reversal learning tasks that require the subject to abandon a previously rewarded strategy following a shift in stimulus–reward mapping (Meunier *et al*. 1997; Shima & Tanji 1998; O'Doherty *et al*. 2001; Kringelbach & Rolls 2003; Hornak *et al*. 2004). Such signals may serve to trigger shifts in LC activation state, thus increasing the likelihood that the animal will leave the current patch to search for a new one.

## 6. Social rewards in primates

In most neurobiological studies of decision making in non-human animals, food or water is delivered for performing a particular action. Such direct and immediate reinforcers are typically referred to as primary rewards, and, as reviewed above, are associated with the activation of midbrain dopamine neurons, as well as neurons in the ventral striatum and OFC, among other areas. In humans, a varied assortment of hedonically positive experiences can evoke activity in these regions, including eating chocolate, hearing pleasant music or even reading a funny cartoon (Blood & Zatorre 2001; Small *et al*. 2001; Mobbs *et al*. 2003; Watson *et al*. 2007).

Although outcomes such as food consumption or the opportunity to mate clearly motivate behaviour, abstract goals such as information gathering or social interaction can also motivate approach or orienting behaviour in the absence of hedonic experience. For primates, in particular, many decisions are motivated by competitive and cooperative interactions with others in a social group (Ghazanfar & Santos 2004). Given the adaptive significance of navigating a complex social environment, one might predict that social stimuli and interactions would evoke activity in neural circuits that overlap with those activated by primary rewards. Indeed, this pattern of activation has been observed in several functional imaging studies. For example, human participants in a ‘prisoner's dilemma’ game show activity in all of the familiar reward-related regions when they engage in bouts of cooperative behaviour with their playing partners: OFC; nucleus accumbens; caudate; and ACC (Rilling *et al*. 2002). The caudate nucleus is also activated when people punish defectors in order to promote cooperation (the so-called ‘altruistic punishment’), even when doing so imposes a personal cost (de Quervain *et al*. 2004).

In many circumstances, images of faces act as potent primary reinforcers and induce neural activity in structures associated with reward processing. For example, the sight of an attractive smiling face activates the medial OFC and the nucleus accumbens (Aharon *et al*. 2001; O'Doherty *et al*. 2003; Ishai 2007). In a classical conditioning experiment, Bray & O'Doherty (2007) demonstrated that an arbitrary visual stimulus acquires value when paired with an attractive face, just as it would when paired with a direct reinforcer such as food. Furthermore, their research confirmed that the neural processes that link the conditioned stimulus with the reward are independent of reward type (e.g. fruit juice, money or an attractive face). Faces may be intrinsically valuable to humans because they direct attention to features of the environment that present information relevant to survival and reproduction. For example, physical features of the face provide information about genetic quality or fertility and thus can be useful in determining whether or not to pursue mating (Jones *et al*. 2001; Soler *et al*. 2003; Roberts *et al*. 2004). In addition to attractiveness, people also use information from faces to assess trustworthiness (Winston *et al*. 2002) and the expected value of cooperation (Singer *et al*. 2004). Together, these observations implicate the operation of a neural system dedicated to linking social stimuli such as faces to the valuation functions guiding behavioural decision making.

Non-human primates also use social information to evaluate their behavioural options. One particularly well-studied aspect of this phenomenon is the use of visual cues to predict the receptivity (Hrdy & Whitten 1987; Waitt *et al*. 2003) or quality (Domb & Pagel 2001) of a potential mate. For example, variations in skin coloration occur in response to hormone levels in both male and female rhesus macaques (Rhodes *et al*. 1997). Female rhesus macaques prefer red male faces over faces with less pigmentation, suggesting that mate choice in this species may be influenced by skin colour (Waitt *et al*. 2003). The reddening of the female rhesus macaque perineum that occurs during oestrus is analogous to the prominent swellings that occur in female chimpanzees and baboons (Dixson 1983; Nunn 1999), providing a potential signal of receptivity and fertility.

Whereas the absence of any obvious analogous signals in human females has led some to suggest that ovulation in our species is a cryptic process, differences in body odour (Singh & Bronstad 2001; Havlicek *et al*. 2006), social behaviour (Matteo & Rissman 1984; Harvey 1987; Haselton *et al*. 2007) and skin coloration (Vandenberghe & Frost 1986) do occur in human females during periods of high fertility. Facial symmetry, a characteristic that both rhesus monkeys and humans find appealing in conspecifics (Rhodes 2006; Waitt & Little 2006), increases in female humans during ovulation (Manning *et al*. 1996). Moreover, such differences are detectable; men find the faces of ovulating women more attractive than those of non-ovulating women (Roberts *et al*. 2004) and pay higher tips for lap dances performed by ovulating women than by menstruating women (Miller *et al*. 2007). Such observations suggest that mate choice in human and non-human primates alike are influenced by ovulatory status via physical and behavioural cues.

Attentiveness to social cues in non-human primates is not limited to the case of mate choice. Studies of primate social behaviour have revealed that monkeys preferentially invest in relationships with dominant individuals (Cheney & Seyfarth 1990; Maestripieri 2007) and are exquisitely sensitive to dominance cues, such as eye contact (Van Hoof 1967). These observations suggest that primate brains compute value functions for specific social and reproductive stimuli that guide behaviour. Deaner *et al*. (2005) explored this hypothesis quantitatively in the laboratory using a pay-per-view task in which male rhesus macaques were given a choice between two targets. Orienting to one target yielded fruit juice but to the other yielded fruit juice and the picture of a familiar monkey. By systematically changing the juice pay-offs for each target and the pools of images revealed, the authors estimated the value of different types of social and reproductive stimuli in a liquid currency.

**Figure 5:**
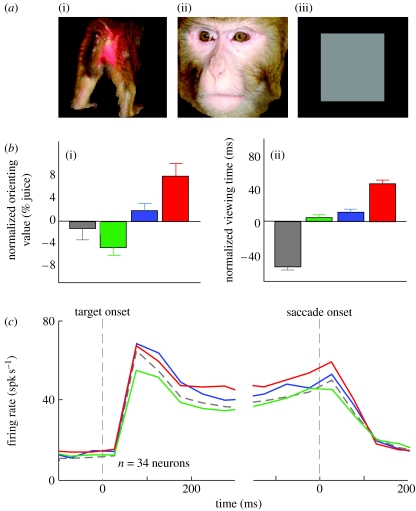
Monkeys value visual signals of status and sex, and parietal cortex signals the value of these images in the visual scene. (*a*) Example images shown to monkeys during a ‘pay-per-view’ task used to assess valuation of socially relevant visual images: (i) female perinea, (ii) monkey faces (high- and low-ranking individuals) and (iii) grey square. (*b*) Mean normalized (i) orienting values and (ii) looking times for various image classes. Orienting values are significantly higher for both the perinea (red bar) and high-status faces (blue bar) in contrast to either the low-status faces (green bar) or grey square (grey bar). Although the monkeys choose to orient more frequently to the high- than low-status faces, the length of time they gaze at either of these image classes are both shorter than the time they spend viewing the perinea. Adapted from Deaner *et al*. (2005). (*c*) Peristimulus time histogram of 34 LIP neurons recorded during the ‘pay-per-view’ task. Note that the activity associated with high-value images, such as female perinea and dominant faces, is consistently greater than that associated with low-value subordinate face images. Adapted from Klein *et al*. (2008). Red line, hindquarters; blue line, dominant; grey line, grey; green line, subordinate.

Their work revealed that male monkeys forego larger juice rewards to view female sexual signals or the faces of high-ranking males, but need overpayment to view the faces of low-ranking males (figure 5). In contrast to the valuation functions governing target choice, the patterns of gaze associated with each class of image hint at the affective complexity associated with social stimuli. Specifically, monkeys looked at female sexual signals for longer than they looked at either high- or low-ranking male faces, perhaps reflecting differences in the hedonic qualities of these stimuli (figure 5).

Several recent studies suggest that some of the same brain areas that mediate valuation of non-social stimuli contribute to valuation of social stimuli as well. For example, a recent study by Rudebeck *et al*. (2006) showed that the ACC is necessary for normal approach and avoidance responses to social stimuli (Rudebeck *et al*. 2006). They measured the latency of macaque monkeys to retrieve a piece of food in the presence of fear inducing stimuli (a rubber snake) or social stimuli (video of other macaques). Unlesioned animals and those with OFC lesions showed normal orienting to the social stimuli, but monkeys with ACC lesions completely ignored them. This result is consistent with the observation that animals with ACC lesions spend less time in the proximity of conspecifics (Hadland *et al*. 2003). Together, these observations indicate that ACC lesions blunt the reinforcing aspects of social interaction.

The observation that monkeys with ACC lesions show reduced orienting to highly salient social stimuli implies that brain areas involved in the control of attention and eye movements, such as parietal cortex, normally receive information about the value of social stimuli from brain areas such as the ACC. This hypothesis was recently tested in a study by Klein *et al*. (2008) who probed the activity of neurons in the LIP area in monkeys performing the pay-per-view task described previously. In this experiment, the target associated with visual outcomes, such as the display of the face of a dominant male or the perineum of a female, was always positioned within the response field of the neuron under study. Klein and colleagues found that LIP neurons responded most strongly when monkeys chose to view images of female sexual signals, less strongly when they chose to view images of the faces of dominant males, and least of all on the rare occasions when they chose to view the faces of subordinate males (figure 5c). These data demonstrate that LIP neurons signal, among other variables, the value of social stimuli in the visual scene. Together, these results endorse the idea that the primate brain is organized, in part, to adaptively acquire valuable social information.

## 7. Economic games

One of the results of the dialogue between biology and economics was the development of evolutionary game theory (Maynard Smith & Price 1973; Smith 1982). As a conceptual framework, game theory can be used to describe the ways in which behaviour is influenced by the behaviour of other animals when competing for limited resources such as mates and food. A classical game describes the interaction of two or more agents with conflicting interests, both trying to maximize some gain. Each game makes precise the number of agents involved, the actions available to those agents and the pay-off that will result from all possible interactions. In economics, the participants in the game identify the costs and benefits available to each player, and are generally expected to adopt a ‘rational’ behavioural strategy. Typically, these behavioural strategies comprise a probabilistic distribution of responses for all players, often called the Nash equilibrium, invulnerable to penetration by other behavioural strategies. In the biological applications of game theory, the economic assumptions of self-interest and rationality are replaced by the evolutionary assumptions of Darwinian fitness and population stability.

In the first direct application of behavioural game theory to neurophysiology, Barraclough *et al*. (2004) studied frequency-dependent decision making in monkeys while recording from neurons in dorsolateral prefrontal cortex (DLPFC). Monkeys played an analogue of matching pennies against a computer opponent. In this game, the animal is rewarded for choosing the target not chosen by the computer. By manipulating the algorithm governing the computer's choices, the experimenters were able to simulate social opponents implementing various strategies. When confronted with an opponent that tracked both the history of choices and rewards received, monkeys' choice frequencies approached the optimal random solution. Neurons in the DLPFC were sensitive to the animal's choice history, the computer's choice history and the value of the rewards on the most recent trials. These signals could in theory be used to update the values of each alternative action, a computation necessary for the animal to choose optimally. This interpretation is consistent with other observations indicating that DLPFC neuron firing reflects the accumulation of sensory evidence during a difficult perceptual discrimination task, as well as the animal's eventual choice (Kim & Shadlen 1999). Functional imaging studies also assign a role for both DLPFC and posterior parietal cortex in decision making in uncertain contexts, particularly as the subject reaches a decision (Huettel *et al*. 2005). These studies imply that DLPFC plays a crucial role in decision making by acting as a comparator of alternative options and then linking the favourable option to the behavioural output.

By contrast, neurons in the dorsal ACC were less likely to encode the actual choice than those in the DLPFC in monkeys playing matching pennies (Seo & Lee 2007). Instead, neurons in the ACC were strongly modulated by rewards received in previous trials (Lee *et al*. 2007). The ability to make strategic behavioural changes in dynamic environments seems likely to require the coordinated interaction of several frontal areas, including the DLPFC, which represents environmental states and the associated behavioural output, the ACC, which represents the outcome of a particular action, and the OFC, which assigns values to particular objects in the environment (Lee *et al*. 2007).

## 8. Conclusion

Although still in the early stages, the union of ethology, economics, psychology and neuroscience—the emerging field of neuroeconomics—offers a potentially powerful way to study the neural mechanisms underlying decision making and behavioural allocation. Just as in other animals, natural selection has shaped human behaviour and its neural substrate. Thus, the behaviour we display today may more strongly reflect the operation of a nervous system that evolved over aeons to optimize hunting and gathering behaviour in small groups rather than to be economically rational (Cosmides & Tooby 1994). These considerations predict that neuroethological studies will be crucial for understanding the neurobiology of reward and decision making in humans as well as other animals.
